# APP family is a regulator of endo-lysosomal membrane vulnerability

**DOI:** 10.1016/j.jbc.2025.110774

**Published:** 2025-09-27

**Authors:** Brianna Lundin, Natalia Wieckiewicz, Midori Yokomizo, Michael Sadek, Desmond Owusu Kwarteng, John R. Dickson, Robert G.R. Sobolewski, Victoria Derosla, Gokce Armagan, Florian Perrin, Bradley T. Hyman, Oksana Berezovska, Masato Maesako

**Affiliations:** MassGeneral Institute for Neurodegenerative Disease, Massachusetts General Hospital, Harvard Medical School, Charlestown, Massachusetts, USA

**Keywords:** APP, endo-lysosome, membrane permeability, lipids, cortex, cerebellum

## Abstract

The amyloid precursor protein (APP) is cleaved by β- and γ-secretases, resulting in the generation of β-amyloid (Aβ). Aβ peptides accumulate in the brain of Alzheimer’s disease, and the removal of toxic Aβ species using antibodies slows the progression of the disease. However, the potential physiological function(s) of APP and its family members remains elusive. Various studies, including ours, reported that APP C99 is primarily processed by γ-secretase in the endo-lysosomal compartments. Here, we report using a series of complementary assays that the endo-lysosomal membrane in APP/amyloid precursor–like protein 2 (APLP2) deficient mouse embryonic fibroblast cells is more vulnerable to leakage caused by oxidative stress, adeno-associated virus, or tau incubation, compared with that in WT controls. The increased vulnerability of the endo-lysosomal membrane is, in part, rescued by APP overexpression, suggesting the contribution of both APP and APLP2. Mechanistically, we observed distinct lipid profiles, including increased cholesterol and Hex1Cer, between the membrane of APP/APLP2 dKO and that of WT mouse embryonic fibroblast cells. Furthermore, we uncovered higher APP expression in primary neurons from the cerebellum of mouse embryos compared with those from the cortex, and the endo-lysosomal membrane in the cerebellum neurons is less vulnerable to leakage than that in the cortical neurons. Taken together, our findings suggest an unrecognized role of APP and its family member in the regulation of endo-lysosomal membrane vulnerability.

The amyloid precursor protein (APP) undergoes proteolytic processing. APP is first processed by α- or β-secretase in extracellular regions, generating APP C-terminal fragments (CTFα/C83 or APP CTFβ/C99) (1, 2, 3, 4, 5). γ-Secretase then cleaves APP C83 or C99 within the membrane, the latter of which results in the production of β-amyloid (Aβ) (6, 7). γ-Secretase repeats C99 cleavage in intervals of three to four amino acids (aa), generating various lengths of Aβ and 3 to 4 aa small peptides (8, 9). There are two other homologs of APP: amyloid precursor–like proteins 1 and 2 (APLP1 and APLP2), which also undergo proteolytic processing by γ-secretase (10, 11). APP family members may serve partially overlapping functions since single APP, APLP1, or APLP2 KO mice show subtle (12, 13), whereas APP/APLP2, APLP1/APLP2 double KO (dKO), and APP/APLP1/APLP2 triple KO mice exhibit lethal phenotypes (14).

Exactly where within the cells γ-secretase cleaves C99 and generates Aβ is one of the crucial questions that remains unclear. Some reports proposed that C99 is processed in the secretory pathway (15, 16, 17), whereas others pointed to the endo-lysosomal compartments (18, 19, 20, 21). Our recent development of the genetically encoded FRET-based biosensor(s) (22, 23) and multiplexed immunocytochemistry has enabled, for the first time, to visually detect the predominant cleavage of C99 by γ-secretase and the generation and enrichment of Aβ in the endo-lysosomal compartments (24, 25). Along the same line, various studies illustrate the link between inefficient C99 processing and endo-lysosomal abnormalities (26, 27, 28, 29, 30, 31). In addition, accumulating evidence highlights the importance of endo-lysosomal membrane integrity in neurodegenerative diseases such as Alzheimer’s disease (AD). For instance, tau endocytosis and the endo-lysosomal membrane permeability play vital roles in tau escape to the cytoplasm (32, 33, 34, 35, 36, 37, 38), which may be one of the critical steps in tau propagation through connected neuronal circuits and tangle formation. Of note, impaired endo-lysosomal membrane integrity is reported to accelerate the spreading of alpha-synuclein (39), suggesting the essential role of the endo-lysosomal membrane in the broader range of neurodegenerative diseases.

It is suggested that γ-secretase predominantly cleaves C99 within the endo-lysosomal membrane (18, 19, 20, 21, 24, 25), and our recent study reported an inverse correlation between endo-lysosomal membrane vulnerability and γ-secretase functioning (40). Hence, this study aims to further determine the role of APP and its family member in endo-lysosomal membrane permeability. First, we employed adeno-associated virus (AAV) transduction, previously validated complementary live-cell imaging, biochemical subcellular fractional assays (40), and split-luciferase complementation analysis of tau endo-lysosomal escape (41) to compare the endo-lysosomal membrane permeability between APP/APLP2-deficient and WT mouse embryonic fibroblast (MEF) cells. Surprisingly, we uncovered that the endo-lysosomal membrane in APP/APLP2 dKO MEF cells is more vulnerable to leakage than WT controls. We also found that tau escape to the cytoplasm is significantly accelerated in the cells lacking APP/APLP2. Interestingly, we uncovered significantly higher APP expression in the mouse cerebellum primary neurons than the cortex neurons, and the cerebellum neurons exhibit decreased endo-lysosomal membrane permeability compared with the cortical neurons. Taken together, our findings shed light on an unrecognized role of APP and its family member in the maintenance of endo-lysosomal membrane integrity.

## Results

### The deficiency of the APP family is associated with increased endo-lysosomal membrane vulnerability to leakage in MEF cells

**Figure 1:**
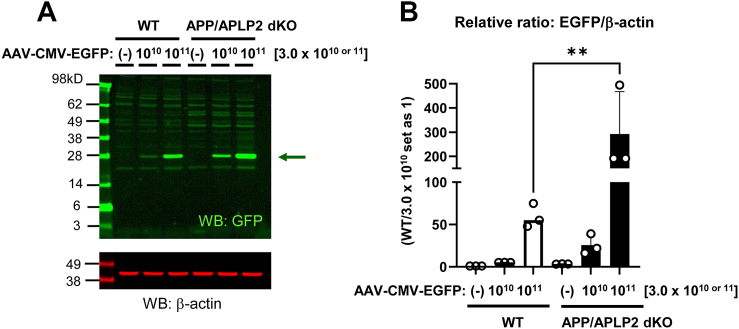
**Increased AAV-mediated EGFP expression in APP/APLP2 dKO MEF cells compared with WT controls.***A,* APP/APLP2 dKO or WT MEF cells were incubated with two different concentrations of AAV–CMV–EGFP (stereotype 8) (3.0 × 10^10^ or 10^11^ GC). Seventy-two hours postincubation, the cells were lysed and subjected to Western blotting using an anti-GFP antibody. β-Actin was used as a loading control. *B,* the band quantification of EGFP over β-actin shows significantly increased EGFP expression in APP/APLP2 dKO compared with WT MEF cells (with 3.0 × 10^10^ GC set as one, and the relative ratios are shown). N = 3 independent experiments. WT *versus* APP/APLP2 dKO, 3.0 × 10^11^, one-way ANOVA. ∗∗*p* < 0.01. AAV, adeno-associated virus; APLP, amyloid precursor–like protein; APP, amyloid precursor protein; CMV, cytomegalovirus; dKO, double KO; EGFP, enhanced GFP; GC, genome copy; MEF, mouse embryonic fibroblast.

Previous studies suggested that γ-secretase predominantly cleaves C99 within the endo-lysosomal membrane (18, 19, 20, 21), which our recent development of novel biosensors has enabled, for the first time, to visualize (24, 25). To further explore the role of the APP family and/or their metabolites in endo-lysosomes, MEF cells from APP/APLP2 dKO mice (42) and WT controls were incubated with different concentrations of AAV packaging the complementary DNA of enhanced GFP (EGFP) for 72 h to gradually transduce the virus into the cells (stereotype 8, 3.0 × 10^10^ or 3.0 × 10^11^ genome copies [GCs]). The lack of Aβ production in APP/APLP2 dKO MEF cells was verified using ELISA (Fig. S1). As expected, EGFP expression increased in both cell lines in a dose-dependent manner. Interestingly, we also found significantly higher EGFP expression in APP/APLP2-deficient cells than in WT controls (Fig. 1, *A* and *B*). Increased EGFP expression in APP/APLP2-deficient cells was also detected following the transduction of stereotype 9 AAV, which packages the same EGFP vector (Fig. S2), suggesting that the effect is not dependent on the specific stereotype used. AAV binds to receptors on the cell surface (43) and is internalized through endocytosis (44, 45). Then, the virus breaks down the endosomal membrane (46), an essential step in delivering its genome into the nucleus. Therefore, we hypothesized that the endo-lysosomal membrane in APP/APLP2-deficient cells is more susceptible to leakage compared with WT controls.

**Figure 2:**
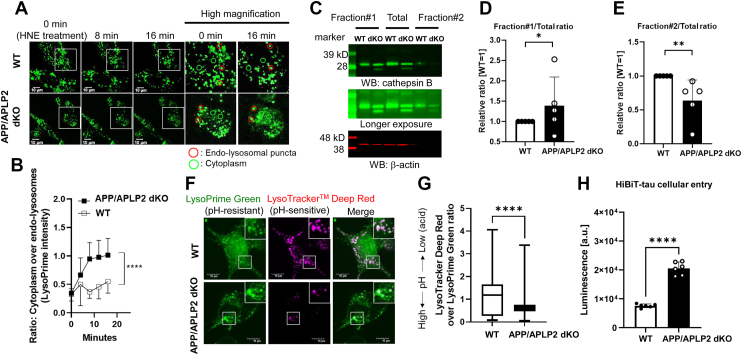
**Complementary assays verify increased endo-lysosomal membrane vulnerability to leakage in APP/APLP2-deficient compared with WT MEF cells.***A,* APP/APLP2 dKO or WT MEF cells were incubated with LysoPrime Green, the fluorescent dye was washed out by HBSS, and the cells were treated with 300 μM HNE to induce endo-lysosomal membrane permeabilization. Changes in LysoPrime Green intensity in the endo-lysosomal puncta (*red circles*) and the cytoplasm (*green circles*) were longitudinally measured for the 20 min imaging period. The scale bar represents 10 μm. *B,* the ratios of LysoPrime Green intensity in cytoplasmic ROIs over endo-lysosomal puncta were more significantly increased over time in APP/APLP2 dKO than in WT MEF cells. N = 18 to 19 ROIs over three to five cells, repeated-measures ANOVA, ∗∗∗∗*p* < 0.0001. The experiment shown is the representative of N = 3 independent experiments. *C,* subcellular fractionation of APP/APLP2 dKO or WT MEF cells using the cytoplasmic extraction buffer (CEB, fraction #1) and membrane extraction buffer (MEB, fraction #2). Parallelly, the cell pellets were lysed using RIPA buffer to create the total fraction. The fractionated samples were subjected to Western blot using an anti-cathepsin B (CTSB) antibody. The ratio of CTSB levels in fraction #1 (CEB) over total fraction was significantly higher (*D*); on the other hand, the ratio in fraction #2 (MEB) over total fraction was lower in APP/APLP2 dKO MEF cells compared with WT controls (*E*), suggesting increased endo-lysosomal membrane permeability in APP/APLP2 dKO MEF cells. N = 5 independent experiments. One-sample *t* test, ∗*p* < 0.05, ∗∗*p* < 0.01. *F,* APP/APLP2 dKO or WT MEF cells were incubated with pH-*resistant* LysoPrime Green and pH-*sensitive* LysoTracker Deep Red, and their emissions were detected using confocal microscopy. The scale bar represents 10 μm. *G,* the ratio of LysoTracker Deep Red emission over that of LysoPrime Green significantly decreased in APP/APLP2 dKO MEF cells compared with WT controls. N = 120 ROIs over six cells. Mann–Whitney *U* test, ∗∗∗∗*p* < 0.0001. The experiment shown is the representative of N = 4 independent experiments. *H,* the luminescence 30 min after 100 nM HiBiT-tau incubation was significantly higher in APP/APLP2 dKO than WT MEF cells. N = 6 biologically independent samples. Unpaired *t* test, ∗∗∗∗*p* < 0.0001. The experiment shown is the representative of N = 4 independent experiments. APLP, amyloid precursor–like protein; APP, amyloid precursor protein; dKO, double KO; HBSS, Hank’s balanced salt solution; HNE, 4-hydroxy-2-nonenal; MEF, mouse embryonic fibroblast; RIPA, radioimmunoprecipitation assay; ROI, region of interest.

To test this hypothesis, we performed live-cell imaging utilizing LysoPrime Green, a pH-insensitive fluorescence dye that labels the endo-lysosomal compartments. In this experiment, APP/APLP2 dKO or WT MEF cells were preincubated with LysoPrime Green, the dye in the culture medium was washed out, and the cells were treated with 4-hydroxy-2-nonenal (HNE, 300 μM) to mimic the condition under oxidative stress and induce endo-lysosomal membrane permeabilization (40, 47). LysoPrime Green intensity in randomly selected regions of interest (ROIs) on endo-lysosomal puncta and cytoplasm was then measured over time (time = 0: right after HNE treatment) (Fig. S3), and the ratio of cytoplasmic over endo-lysosomal fluorescence intensity was compared between the two cell lines (Fig. 2*A*) (40). We found that the ratio of LysoPrime Green intensity in cytoplasmic ROIs over endo-lysosomal puncta was increased over time more significantly in APP/APLP2 dKO MEF cells than in WT controls (Fig. 2*B*), suggesting that the endo-lysosomal membrane in the cells that lack APP and APLP2 is more vulnerable to HNE treatment than WT MEF cells.

To validate the findings from the LysoPrime Green live-cell imaging, we next performed subcellular fractionation, followed by Western blotting to measure the levels of cathepsin B (CTSB), a lysosomal hydrolase. In this complementary biochemical approach, APP/APLP2 dKO and WT MEF cells were suspended in PBS, the cell pellets from half of the suspension were solubilized in cytoplasmic extraction buffer (CEB) to create fraction #1, whereas the CEB-insoluble pellets were lysed with membrane extraction buffer (MEB) to create fraction #2. The other half of the suspension was solubilized with radioimmunoprecipitation assay (RIPA) buffer to obtain the total fraction. CTSB levels in fraction #1 over RIPA buffer–extracted total fraction and those in fraction #2 over total fraction serve as indicators of endo-lysosomal membrane vulnerability (40). We found that the ratio of CTSB in fraction #1 over the total fraction is significantly higher in APP/APLP2 dKO than in WT MEF cells (Fig. 2, *C* and *D*). On the other hand, APP/APLP2 dKO MEF cells exhibit lower levels of CTSB in the MEB fraction #2 compared with WT controls; thus, the ratio of CTSB in fraction #2 over the total fraction was significantly lower in APP–APLP2-lacking cells (Fig. 2, *C* and *E*). These findings further support the conclusion that endo-lysosomal membrane in APP/APLP2 dKO MEF cells is more vulnerable compared to WT controls.

Next, we coincubated APP/APLP2 dKO or WT MEF cells with LysoTracker Deep Red (pH-*sensitive*) and LysoPrime Green (pH-*resistant*), to semiquantitatively compare endo-lysosomal pH in live cells using confocal microscopy (Fig. 2*F*). For the analysis, ROIs were randomly generated on LysoPrime Green–positive puncta, and then fluorescence intensities of LysoTracker Deep Red and LysoPrime Green were measured in individual ROIs. We found thatthe ratio of LysoTracker Deep Red over LysoPrime Green intensities was significantly lower in APP/APLP2 dKO compared with WT MEF cells (Fig. 2*G*), indicating that APP/APLP2 dKO MEF cells exhibit higher endo-lysosomal pH than WT controls.

Accumulating evidence demonstrates that tau is endocytosed (34, 36, 38), and the endo-lysosomal membrane could be the gateway of tau cellular access/entry (32, 33, 35, 37). Therefore, we lastly examined if tau cytoplasmic access is accelerated in APP/APLP2 dKO MEF cells compared with WT controls using a split luciferase complementation assay (41). In this assay, a large 18 kD unit of the deep-sea shrimp–derived luciferase (LgBiT) fused to beta-actin was expressed in the cytoplasm of APP/APLP2-deficient or WT cells. Then, the cells were incubated for 30 min with recombinant 2N4R tau proteins fused to 11 aa high-affinity peptide at the amino terminus (HiBiT-tau, 100 nM). The cell-entered HiBiT-tau binds to the LgBiT, which results in the complementation of luciferase activity and luminescence in the presence of substrate (41). We found that the luminescence was significantly higher in APP/APLP2 dKO MEF cells compared with WT controls (Fig. 2*H*). Next, to measure total intracellular HiBiT-tau levels, which include HiBiT-tau proteins that are endocytosed and localized within the endo-lysosomal compartments and those escaped from the endo-lysosomes and localized in the cytoplasm, we incubated WT or APP/APLP2 dKO MEF cells with 100 nM HiBiT-tau for 30 min. After the incubation, the conditioned medium containing HiBiT-tau was washed out, and cell lysates were subjected to the Nano-Glo HiBiT Lytic Detection System, in which the amount of HiBiT-tau in cells can be determined by adding a lytic detection reagent containing the substrate and LgBiT. While the total taken-up levels were also significantly higher in APP/APLP2 dKO cells than in WT controls (Fig. S4*A*), the percent of endo-lysosomal escape, which is calculated by dividing the amount of escaped HiBiT-tau (Fig. 2*H*) by the total intracellular HiBiT-tau levels (Fig. S4*A*), is still significantly higher in APP/APLP2 dKO compared with WT MEF cells (Fig. S4*B*). This suggests that HiBiT-tau cytoplasmic entry is significantly increased in the cells lacking APP and APLP2. Collectively, our results from a wide range of experiments strongly support the hypothesis that the endo-lysosomal membrane in APP/APLP2 dKO MEF cells is more vulnerable to permeabilization than that in WT controls.

We further aimed to address whether the vulnerable endo-lysosomal membrane phenotype is associated with APP. As such, APP/APLP2 dKO MEF cells were transiently transfected with either empty vector or WT APP V5. Twenty-four hours after transfection, the cells were incubated with AAV–cytomegalovirus (CMV)–EGFP for 48 h (stereotype 8, 3.0 × 10^11^ GC), and EGFP expression was assessed using Western blotting. We found that EGFP levels in WT APP V5–transfected cells were significantly decreased compared with those in cells transfected with an empty vector. Moreover, EGFP expression was not fully suppressed by APP expression (Fig. S5, *A* and *B*), indicating that not only APP but also APLP2 plays a role in maintaining endo-lysosomal membrane integrity.

### Altered membrane lipid profiles in the cells lacking the APP family proteins

**Figure 3:**
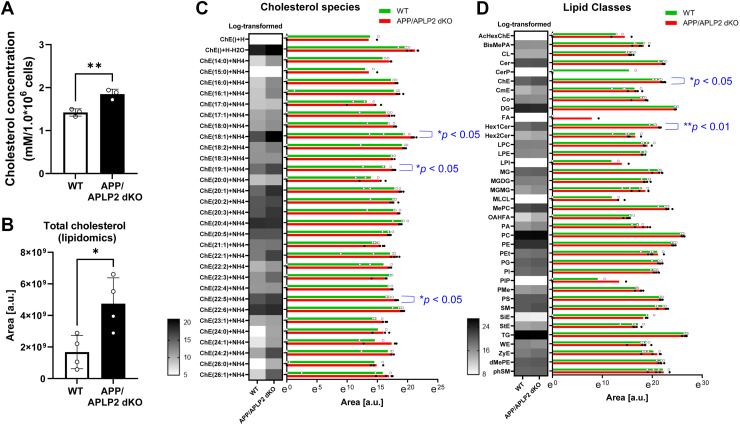
**Comprehensive lipid profiles in the membrane fraction of APP/APLP2 dKO MEF cells and WT controls.***A,* the same number of APP/APLP2 dKO or WT MEF cells was subjected to subcellular fractionation, and total cholesterol levels in their membrane fractions were measured using the total cholesterol colorimetric assay kit. Total cholesterol levels significantly increased in APP/APLP2 dKO MEF cells compared with WT controls. N = 3. Unpaired *t* test, ∗∗*p* < 0.01. *B,* the membrane fraction of APP/APLP2 dKO MEF cells or WT controls was subjected to LC–MS/MS lipidomic analysis, and the signal area (a.u.) was measured for various lipid species (N = 4 independent experiments). The analysis recapitulated the significantly increased total cholesterol level, which is based on the sum of all the detected cholesterol species, in APP/APLP2 dKO compared with WT MEF cells. Unpaired *t* test, ∗*p* < 0.05. *C,* the heatmap and bar graphs were generated from log-transformed area and the exact area data, respectively, showing differences in individual cholesterol species between APP/APLP2 dKO MEF cells and WT controls. *Dots* in bar graphs correspond to the values in individual experiments, and *bar graphs* show means. Mann–Whitney *U* test, ∗*p* < 0.05. *D,* similarly, the heatmap and bar graphs are shown for other lipid species, where the areas of all the lipid species are combined. Mann–Whitney *U* test, ∗∗*p* < 0.01, ∗*p* < 0.05. APLP, amyloid precursor–like protein; APP, amyloid precursor protein; dKO, double KO; MEF, mouse embryonic fibroblast.

To investigate the effect of lacking APP family proteins on membrane lipids, we extracted the membrane fraction from APP/APLP2 dKO or WT MEF cells and measured cholesterol levels using a colorimetric assay. Similar to what was reported previously (48), we found significantly higher cholesterol levels in the membrane fraction of APP/APLP2 dKO MEF cells compared with those of WT controls (Fig. 3*A*). To further verify this finding, we performed LC–MS/MS-based lipidomic analysis. Consistent with the result from the colorimetric assay, we found significantly increased total cholesterol levels in the membrane fraction of APP/APLP2 dKO MEF cells compared with that of WT controls (Fig. 3*B*). Many cholesterol subclasses showed an increased trend in APP/APLP2 dKO MEF cells compared with WT controls, and ChE(18:1) + NH4, ChE(19:1) + NH4, and ChE(22:5) + NH4 demonstrated statistically significant differences (Fig. 3*C*). In addition to cholesterol, we found that Hex1Cer is also elevated in the membrane of APP/APLP2 dKO MEF cells in a statistically significant manner (Fig. 3*D*). These results point to a possible mechanistic link between altered lipid profiles and increased membrane vulnerability in APP/APLP2 dKO MEF cells.

### Higher APP expression and decreased endo-lysosomal membrane vulnerability in the cerebellum compared with the cortex-derived primary neurons

**Figure 4:**
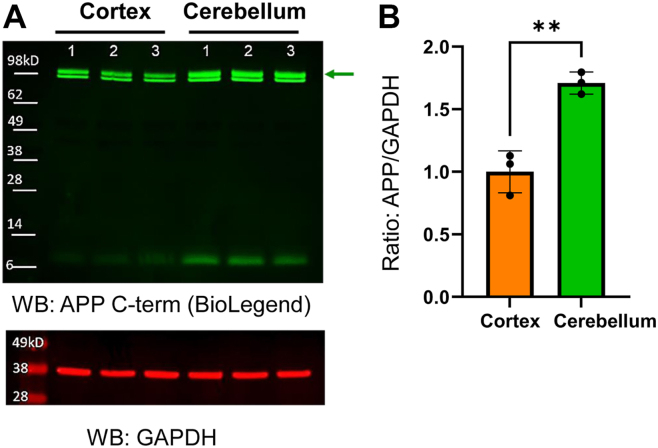
**Higher APP expression in the cerebellum than in the cortex-derived primary neurons.***A,* Western blot analysis using anti-APP mouse monoclonal and GAPDH (loading control) antibodies. *B,* showing that APP expression is significantly higher in the cerebellum than in the cortical primary neurons. N = 3, unpaired *t* test, ∗∗*p* < 0.01. APP, amyloid precursor protein.

Since tau pathology is predominant in the cortical regions of AD brains while barely in the cerebellum (49, 50), primary neurons were dissociated from the cerebellum or cortex of the same embryos and cultured for 12 to 15 days *in vitro* (Fig. S6, *A* and *B*). Interestingly, we found significantly higher APP expression in the cerebellum than in the cortex neurons (Fig. 4, *A* and *B*). Our cultures were stained with neuronal (*i.e.*, MAP2) and non-neuronal markers (*i.e.*, GFAP and IBA1) (Fig. S7*A*). We confirmed that the majority of cells are MAP2-positive neurons (Fig. S7*B*), which is comparable between the cerebellum and cortex cultures (Fig. S7*C*). In addition, the higher APP expression in the cerebellum compared with the cortical neurons was verified by a different APP polyclonal antibody (Fig. S8, *A* and *B*). Lastly, we compared APP expression between the cerebellum and cortex of (1) an adult mouse, (2) an embryo, or (3) cultured primary neurons and found that the neurons in culture show a clear difference in APP expression between the two brain regions (Fig. S8*C*).

**Figure 5:**
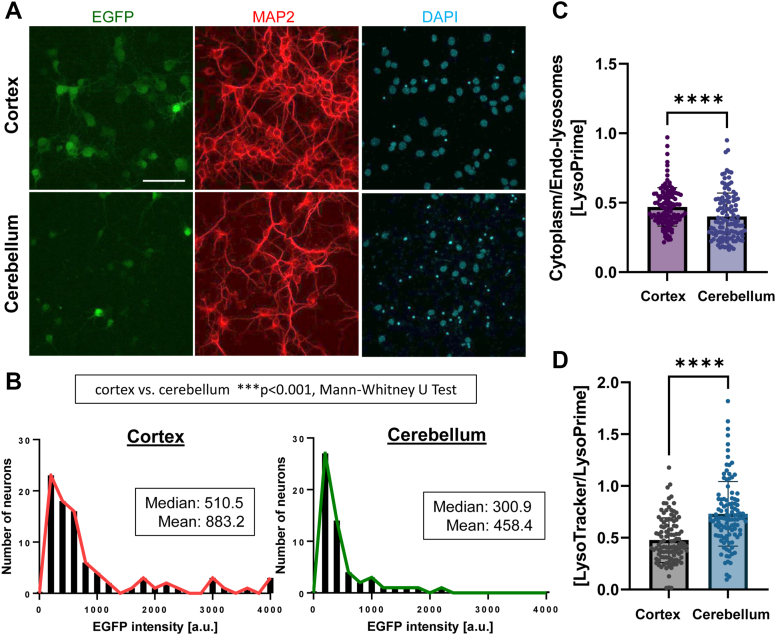
**Decreased endo-lysosomal membrane vulnerability to leakage in the cerebellum than in the cortex neurons.***A,* the cerebellum or cortical-derived primary neurons were incubated with AAV–CMV–EGFP (stereotype 8) (3.0 × 10^11^ GC) for 72 h and stained with an MAP2 and Cy3-conjugated secondary antibodies. The scale bar represents 60 μm. *B,* EGFP fluorescence intensity in MAP2-positive neurons was measured by confocal microscopy. The histogram (*Y*-axis: number of neurons, *X*-axis: EGFP intensity) shows that the cerebellum neurons express lower levels of EGFP compared with the cortical neurons. N = 55 to 85, Mann–Whitney *U* test, ∗∗∗*p* < 0.001. The experiment shown is the representative of N = 5 independent experiments. *C,* the ratios of LysoPrime Green intensity in cytoplasmic ROIs over endo-lysosomal puncta post 300 μM HNE for 20 min were significantly lower in the cerebellum than in the cortical neurons. N = 108 to 110 ROIs from three to five different neurons, Mann–Whitney *U* test, ∗∗∗∗*p* < 0.0001. The experiment shown is the representative of N = 4 independent experiments. *D,* the ratio of LysoTracker Deep Red emission over that of LysoPrime Green was significantly increased in the cerebellum compared with the cortical neurons. N = 110 ROIs from nine neurons. Mann–Whitney *U* test, ∗∗∗∗*p* < 0.0001. The experiment shown is the representative of N = 3 independent experiments. AAV, adeno-associated virus; CMV, cytomegalovirus; EGFP, enhanced GFP; GC, genome copy; HNE, 4-hydroxy-2-nonenal; ROI, region of interest.

Since APP expression is significantly higher in the cerebellum than in the cortex neurons, we asked if the cerebellum neurons exhibit decreased endo-lysosomal membrane vulnerability compared with the cortex neurons. To address this, the cerebellum or cortex primary cultures were incubated with AAV–CMV–EGFP (stereotype 8, 3.0 × 10^11^ GC) for 96 h. Immunostaining with MAP2 and Cy3-conjugated secondary antibodies was performed to identify neurons, and EGFP fluorescence intensity in MAP2-positive neurons was measured by confocal microscopy (Fig. 5*A*). Strikingly, we found that the cerebellum neurons express significantly lower EGFP levels than the cortical neurons (Fig. 5*B*). Next, the cerebellum and cortex neurons were incubated with LysoPrime Green, followed by the treatment with 300 μM HNE for 20 min to induce endo-lysosomal membrane permeabilization (40, 47). We found a significantly lower ratio of cytoplasmic over endo-lysosomal fluorescence intensity in the cerebellum than in the cortical neurons (Fig. 5*C*), suggesting less leakage of LysoPrime Green post-HNE treatment in the cerebellum compared with the cortical neurons. Moreover, we found that overexpression of APP in cortical neurons significantly decreases the LysoPrime Green cytoplasmic over endo-lysosomal ratios compared with neurons expressing a control empty vector (Fig. S9, *A* and *B*). Lastly, we found the ratio of LysoTracker Deep Red (pH-sensitive) over LysoPrime Green (pH-resistant) intensities was significantly higher in the cerebellum than in the cortical neurons (Fig. 5*D*), suggesting that the cerebellum neurons exhibit lower endo-lysosomal pH than the cortical neurons. Taken together, these data strongly suggest that the endo-lysosomal membrane in cerebellum primary neurons, which exhibit higher APP expression, is less vulnerable to permeabilization than the cortical neurons.

## Discussion

Numerous studies, including ours, suggest that γ-secretase processes C99 and generates Aβ primarily in the endo-lysosomal compartments (18, 19, 20, 21, 24, 25). Here, we show that the endo-lysosomal membrane in APP/APLP2 dKO MEF cells is more vulnerable to leakage than WT controls (Figs. 1 and 2). Furthermore, the cerebellum primary neurons exhibit higher APP expression and decreased endo-lysosomal membrane vulnerability compared with the cortical neurons (Figs. 4 and 5). These results highlight an unrecognized role of APP and its family member in the maintenance of endo-lysosomal membrane integrity.

Our present study reports a novel link between APP family proteins and endo-lysosomal membrane vulnerability to leakage; however, the underlying mechanism remains elusive. APP forms a complex with FE65 and lipoprotein receptors, such as ApoER2 (51) and very low-density lipoprotein receptor (52), regulating their cell surface expression. Therefore, the abnormal regulation of lipoprotein receptors localization and corresponding lipid ligand endocytosis as well as exocytosis could be one of the mechanisms underlying the increased endo-lysosomal membrane permeability in APP defcient cells. Indeed, we found significantly different cholesterol and Hex1Cer levels in the membrane fraction of APP/APLP2 dKO MEF cells (Fig. 3). Primary neurons from the cortex and cerebellum suggest that a 40% reduction in APP levels (Fig. 4) is associated with increased AAV transduction (Fig. 5, *A* and *B*), leakage of LysoPrime into the cytoplasm (Fig. 5*C*), and elevated endo-lysosomal pH (Fig. 5*D*). Therefore, there could be a window of APP expression that is required for the endo-lysosome membrane homeostasis.

On the other hand, previous studies found that the activities of the enzymes responsible for cholesterol biosynthesis are regulated by APP (53) and/or Aβ (48). Moreover, cholesterol-binding sites are identified on the juxtamembrane as well as transmembrane regions of APP (54), which are conserved within C99 and Aβ sequences. Of note, the juxtamembrane and transmembrane regions of APLP2 have some degree of amino acid overlap with the same APP regions. Similar to a previous report (48), we found, using an enzyme-coupled reaction assay and mass spectrometry lipidomic analysis in which cell numbers were carefully adjusted, that the level of total cholesterol is significantly increased in the membrane of APP/APLP2 dKO MEF cells compared with that of WT controls (Fig. 3). Therefore, it is plausible that cholesterol plays a pivotal role in regulating endo-lysosomal membrane vulnerability to leakage. However, a previous study reported that astrocytes lacking APP exhibit reduced cholesterol levels (55). This controversy warrants further investigations to establish clear causative relationships among cholesterol, the APP family, and endo-lysosomal membrane vulnerability.

In addition, cells have several strategies to repair damaged endo-lysosomal membrane. For example, these include the recruitment of the endosomal sorting complexes required for transport machinery (56, 57) and annexins (58, 59) to the damaged sites, the formation and recruitment of stress granules to the damaged sites (60), the formation of ceramide microdomains around the damaged sites (61), and the supply of lipids from endoplasmic reticulum *via* a number of lipid transfer enzymes that include oxysterol-binding protein 1 (OSBP) and the OSBP-related proteins (ORPs) (62). Thus, the other possibility is that APP, APLP2, and/or their metabolites may be associated with these key factors, interfering with the repair process of the damaged endo-lysosomal membrane. For example, a recent study has demonstrated that Aβ is responsible for the formation of stress granules (63). The lack of APP may slow endo-lysosomal membrane repair *via* less efficient formation of stress granules.

Protein aggregates, such as tau, are known to spread from a neuron to the neighboring neurons in neurodegenerative diseases (review in Ref. (64)). How aggregates access the cytosol after their release into the extracellular space is actively investigated, and endocytosis has recently gained more attention. For example, tau binds to heparin sulfate proteoglycans (65) and LRP1 (36) at the neuronal surface, being delivered to the endo-lysosomal compartments. Furthermore, CRISPR interference screenings uncovered several essential molecules localized within the endo-lysosomal pathway as molecular regulators of tau propagation (33). Moreover, it is reported that tau seed–containing exosomes are endocytosed and mediate the escape of the tau aggregates into the cytoplasm (35). In addition, impaired endo-lysosomal membrane integrity is reported to accelerate alpha-synuclein spreading as well (39), signifying the essential role of the endo-lysosomal membrane in the broader range of neurodegenerative diseases. Our discovery of the novel role of the APP family in the endo-lysosomal membrane integrity may provide a new model to determine the exact gateway of protein aggregates’ cellular entry.

While tau pathology is widely detected in the cortical areas, it is hardly seen in the cerebellum of the AD brain (49, 50). One of the potential underlying mechanisms could be the lower expression of total and phosphorylated tau in the cerebellum than in the cortex (66). Establishing the molecular signatures of primary neurons derived from the cortex and the cerebellum may, in part, help to understand the mechanism underlying brain regional vulnerability to tau pathology. Moreover, AAV-mediated gene delivery has been primarily used to express proteins of interest in cells. However, postbinding to receptors on the cell surface, such as heparin sulfate proteoglycans (43), and internalization through endocytosis (44, 45), AAV is known to break down the endosomal membrane (46) to deliver its genome into the nucleus. The cell entry process of the virus is somewhat relevant to how misfolded proteins travel from one neuron to the neighboring neurons. Not only can virus biology be adapted to study how misfolded proteins are spread, but the extensive knowledge in the field may also provide a clue to elucidate the cause-and-effect relationship between misfolded protein propagation and neurodegeneration. Of note, it has been reported that lowering membrane fluidity by serum starvation or cholesterol treatment inhibits adenovirus entry (67).

It is worthwhile to note that APP/APLP2-deficient cells exhibit elevated endo-lysosomal pH. However, the underlying molecular mechanisms remain unclear. Endo-lysosomal pH can significantly increase because of the rupture and/or damage of the endo-lysosomal membrane (68). While the leakage of LysoPrime Green from endo-lysosomal compartments to the cytoplasm post-HNE treatment was more significant in APP/APLP2 dKO than in WT MEF cells (Fig. 2, *A* and *B*), such leakage was not detected at the baseline (*i.e.*, without HNE treatment) (Fig. S3*A*). This suggests that APP/APLP2-deficient cells do not exhibit endo-lysosomal membrane breaks, rather highlighting the other possible mechanisms, such as decreased V-ATPase activity and/or increased activities of H^+^ channels such as TMEM175. It is reported that C99 binds within a pocket of the V-ATPase V0a1 subunit cytoplasmic domain and competitively inhibits the association of the V1 subcomplex, resulting in reduced V-ATPase activity (28). On the other hand, a previous study uncovered an interaction between Aβ and TMEM175 (69). Since both hydrogen ions and Aβ are localized in the lumen of endolysosomes, the effect of Aβ on TMEM175 activity would be keen to be further investigated.

In summary, this study reports a role of APP family proteins in the regulation of endo-lysosomal membrane vulnerability. Whether the increased endo-lysosomal membrane vulnerability observed in APP/APLP2 dKO MEF cells results from loss of APP/APLP2 processing by γ-secretase remains an open question to be addressed. RNAi therapeutic silencing of APP has recently moved to clinical trials for AD and cerebral amyloid angiopathy. Our findings would be important to consider the benefits and drawbacks of the silencing approach and warrant further investigation.

## Experimental procedures

### Antibodies, reagents, plasmid DNA, AAV, and lentivirus

Anti-APP C-terminal antibodies were purchased from BioLegend and MilliporeSigma. Anti-β-actin and GFAP antibodies were from MilliporeSigma; anti-CTSB antibody was from Abcam; anti-GFP and anti-V5 tag antibodies were from Thermo Fisher Scientific; anti-presenilin 1 (PSEN1), anti-presenilin 2 (PSEN2), anti-MAP2, and anti-GAPDH antibodies were from Cell Signaling Technology, Inc; and anti-IBA1 antibody was from FUJIFILM Wako Chemicals. Anti-LAMP1 antibodies were from Abcam and MilliporeSigma.

LysoPrime Green was purchased from Dojindo Molecular Technologies, Inc; LysoTracker Deep Red was from Thermo Fisher Scientific; and HNE and dimethyl sulfoxide were from MilliporeSigma.

WT APP V5 plasmid was developed in a previous study (19). The AAV packaging of the complementary DNA of EGFP under the CMV promoter (stereotype 8: 3.13 × 10^13^, stereotype 9: 2.40 × 10^13^ GC/ml) was purchased from Addgene. Plasmid vector development and the lentivirus packaging of pLV[Exp]-CBA-LgBiT-beta-actin (70) (3.33 × 10^8^ TU/ml), pLV[Exp]-SYN1>mouse APP (2.88 × 10^8^ TU/ml), and its empty control vector (4.02 × 10^8^ TU/ml) were performed by VectorBuilder, Inc.

### Tissue culture and transfection

WT and APP–APLP2 dKO (42) MEFs were cultured in Opti-MEM Reduced Serum Medium (Thermo Fisher Scientific) with 5% fetal bovine serum (Atlanta Biologicals, Inc). The cells were authenticated using short tandem repeat profiling and monitored for mycoplasma contamination. Lipofectamine 3000 (Thermo Fisher Scientific) was used for transient transfection according to the manufacturer’s instructions.

Primary neurons were dissociated from the cortex or the cerebellum of CD1 mouse embryos at E14 to 16 (Charles River Laboratory) using the Papain Dissociation System (Worthington Biochemical Corp), and cultured in Neurobasal medium plus 2% B27 supplement, 1% GlutaMAX, and 1% penicillin–streptomycin (Thermo Fisher Scientific) for 12 to 15 days *in vitro*. The animal protocol was approved by the Massachusetts General Hospital Animal Care and Use Committee (#2003N000243).

### Aβ ELISA

Aβ40 level was measured using the Human/Rat Beta-Amyloid (40) ELISA kit (FUJIFILM Wako Chemicals USA Corporation) according to the manufacturer’s instructions.

### Subcellular fractionation and Western blotting

The CEB containing protease and phosphatase inhibitor cocktail in the Subcellular Protein Fractionation Kit for Cultured Cells (Thermo Fisher Scientific) was added to the pellet of MEF cells, followed by gentle pipetting five times to lyse the cells. The lysed cells were incubated at 4 °C for 10 min with gentle rotation. Then, the cell lysates were centrifuged at 500*g* for 5 min, and the supernatants were immediately transferred to clean tubes (fraction #1). The insoluble pellets were lysed using the MEB, vortexed for 15 s, and incubated at 4 °C for 10 min with gentle rotation. Then, the lysates were centrifuged at 3000*g* for 5 min, and the supernatants were transferred to clean tubes (fraction #2). To obtain the total fraction, RIPA buffer (Sigma–Aldrich) containing protease and phosphatase inhibitor cocktail was used to lyse the cells, followed by incubation on ice for 30 min. After centrifuging at 14,000*g* for 20 min, the supernatant was used as the total fraction.

Protein concentration was measured using the BCA Protein Assay kit (Thermo Fisher Scientific). The samples were mixed with NuPAGE LDS Sample Buffer and NuPAGE Sample Reducing Agent (Thermo Fisher Scientific). After boiling, the samples were subjected to SDS-PAGE on NuPAGE 4% to 12% Bis–Tris Protein gels (Thermo Fisher Scientific), followed by transfer to nitrocellulose membranes (Thermo Fisher Scientific) using the iBlot 2 Gel Transfer Device (Thermo Fisher Scientific) or Bio-Rad Wet electroblotting system (Bio-Rad). The membranes were incubated with primary and corresponding fluorophore-conjugated secondary antibodies and developed using the LI-COR Odyssey CLx scanner digital imaging system (LI-COR Biosciences).

### Immunocytochemistry and confocal microscopy

Neurons transduced with AAV–CMV–EGFP were fixed with 4% paraformaldehyde (VWR International), washed by PBS, and permeabilized by 0.1% Triton X-100 + 1.5% normal donkey serum (Jackson ImmunoResearch, Inc) for 1 h. The neurons were then incubated with primary antibodies overnight, Cy3-conjugated secondary antibodies for 1 h, and mounted with a coverslip using VECTASHIELD Antifade Mounting Medium with 4′,6-diamidino-2-phenylindole (Vector Laboratories).

An Olympus FV3000RS Confocal Laser Scanning Microscope was used to detect fluorescence. The scope was equipped with a CO_2_/heating unit (Tokai-Hit) to maintain suitable CO_2_ concentration and heating for live-cell imaging. Moreover, the TruFocus Z drift compensation module was used to maintain focus during time-lapse imaging. A 40×/0.95 numerical aperture objective was used for image acquisition. Lasers at 405 nm, 488 nm, and 561 nm were used to excite 4′,6-diamidino-2-phenylindole, EGFP, and Cy3, and their emissions were detected within 430 to 470 nm, 490 to 540 nm, and 560 to 620 nm, respectively. A laser at 488 nm was used for the excitation of LysoPrime Green, and emission was detected within 500 to 540 nm. A laser at 640 nm was used to excite LysoTracker Deep Red, and its emission was detected within 670 to 770 nm. ImageJ was used to measure fluorescent intensity in ROIs.

### A split luciferase complementation tau assay

To assess cytoplasmic entry of tau, WT, APP/APLP2 dKO MEF cells, or primary neurons were cultured in 96-well plates and transduced with the lentiviral particles packaging pLV[Exp]-CBA-LgBiT-beta-actin. Two days (for MEF cells) or 4 days (for neurons) post-transduction, the cells were incubated with 100 nM 2N4R HiBiT-tau for 30 min at 37 °C. The medium was washed out, and the cells were incubated with Nano-Glo Live Cell Substrate in Nano-Glo Live Cell Assay System (Promega Corporation). The luminescence was measured using the Wallac 1420 victor^2^ multilabel counter (PerkinElmer).

To measure total intracellular HiBiT-tau levels, including those endocytosed and localized within the endo-lysosomal compartments, and those that escaped from endo-lysosomes and invaded the cytoplasm, the Nano-Glo HiBiT Lytic Detection System (Promega Corporation) was used.

### A colorimetric total cholesterol assay and lipidomic analysis

To measure total cholesterol levels, including free cholesterol and cholesterol esters, the total cholesterol colorimetric assay kit (Elabscience) was used according to the manufacturer’s protocol.

Lipidomic analysis was performed at the Beth Israel Deaconess Medical Center Mass Spectrometry Core, as described previously (71). After the cell number was equalized, the membrane fractions from APP–APLP2 dKO or WT MEF cells were subjected to methanol:chloroform 2:1 (v/v). After chloroform and water were added, the mixture was agitated and vortexed. Then, the mixture was centrifuged at 3500 rpm to separate the two phases (72), and the lower chloroform phase containing lipids was evaporated and analyzed *via* LC–MS/MS using the QExactive Plus/HF Orbitrap HR-LC–MS/MS Spectrometer and LipidSearch software (ThermoFisher Scientific). The area size of the signal peak was measured in four independent experiments to perform statistical analysis. Notably, some lipids were under detection in some experiments, whose values are set as 1. This enables us to indicate their undetectability in graphs and perform statistical analysis while preventing false positivity.

### Statistical analysis

GraphPad Prism 9 (GraphPad Software) was used to perform statistical analysis. The D’Agostino and Pearson's omnibus normality test was used to examine the Gaussian distribution of the data and the variance equality. Unpaired *t* tests, one-sample *t* tests, Mann–Whitney *U* tests, one-way, two-way, and repeated-measures ANOVA were used to compare the data. At least three independent experiments were performed to ensure the reproducibility of the results.

## Data availability

All data are contained within the article.

## Supporting information

This article contains supporting information.

## Conflict of interest

The authors declare that they have no conflicts of interest with the contents of this article.

## Supporting information

Supplmental Figures
