# Supplementary material for: APP family is a regulator of endo-lysosomal membrane vulnerability
Source: J Biol Chem. 2025 Sep 27;301(11):110774. doi: 10.1016/j.jbc.2025.110774 (PMC12605294; doi:10.1016/j.jbc.2025.110774)
Supplement: Supplmental Figures [file mmc1.pdf]

Supporting information

APP family is a regulator of endo-lysosomal membrane permeability

Authors: Brianna Lundin[1#], Natalia Wieckiewicz[1#], Midori Yokomizo[1], Michael Sadek[1], Desmond Owusu Kwarteng[1], John R Dickson[1], Robert GR Sobolewski[1], Victoria Derosla[1], Gokce Armagan[1], Florian Perrin[1], Bradley T Hyman[1], Oksana Berezovska[1], and Masato Maesako[1]*

[1]MassGeneral Institute for Neurodegenerative Disease, Massachusetts General Hospital, Harvard Medical School, 114, 16th street, Charlestown, MA 02129

[#]Equal contribution
*Correspondence: Masato Maesako, PhD, Email: MMAESAKO@mgh.harvard.edu

Supplemental Figure 1

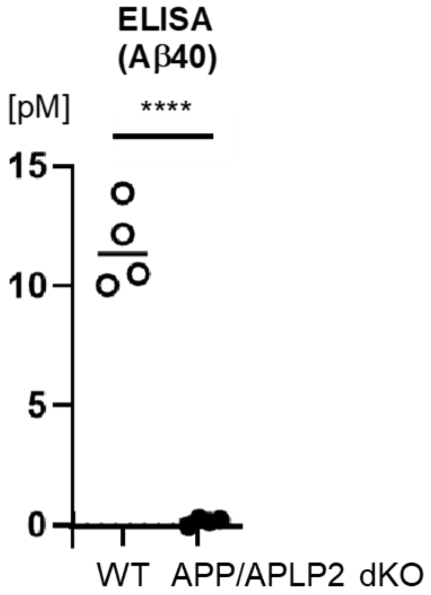

ELISA shows abolished generation of Aβ40 in APP/APLP2 dKO MEF cells. N = 4 independent biological samples, Unpaired t-test, ****$p<0.0001$.

Supplemental Figure 2

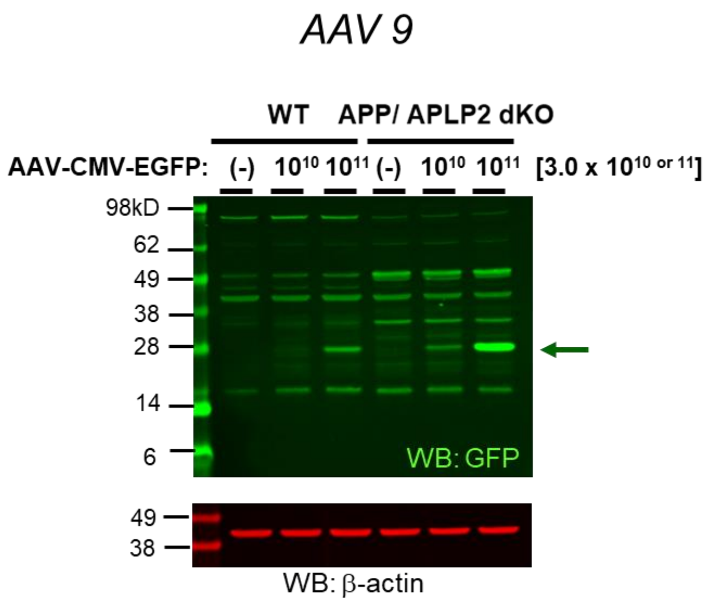

Increased EGFP expression in APP-deficient MEF cells compared to WT controls post-incubation of *stereotype 9* AAV-CMV-EGFP (3.0 x 10^{10} or 10^{11} GC).

Supplemental Figure 3

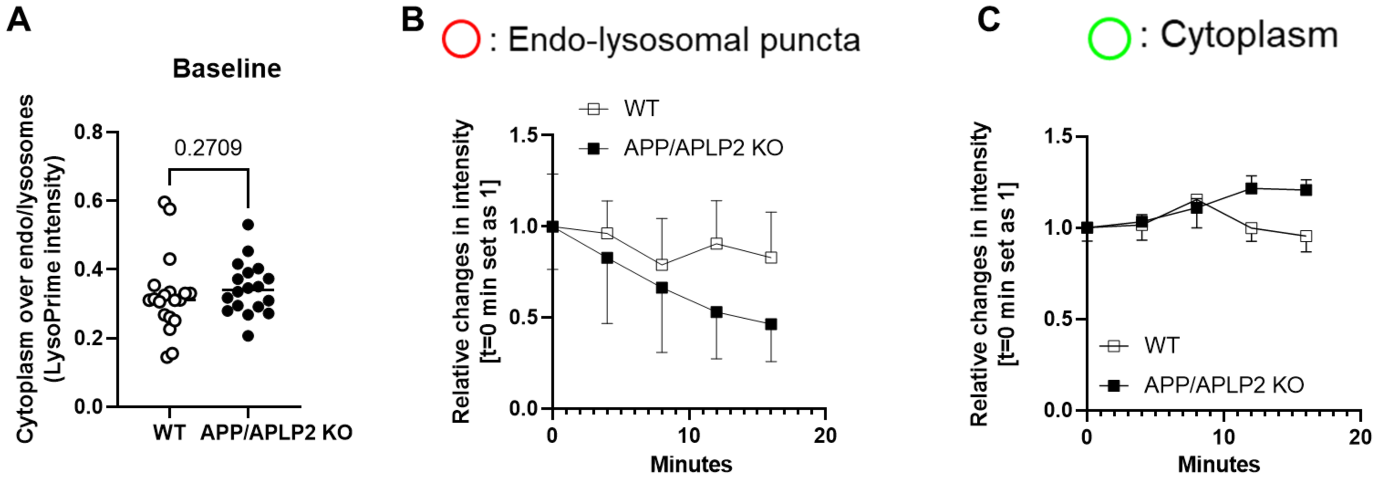

APP/APLP2 dKO MEF cells or WT controls were incubated with LysoPrime Green, the fluorescent dye was washed out by HBSS, and the cells were treated with 300 μM HNE to induce endo-lysosomal membrane permeabilization. **(A)** The ratio of LysoPrime Green intensity in the cytoplasmic ROIs over endo-lysosomal puncta is not different between APP/APLP2 dKO MEF cells and WT controls before HNE treatment (N = 18-19 ROIs over 3-5 cells). Longitudinal changes in LysoPrime Green intensity in the endo-lysosomal puncta **(B)** and the cytoplasm **(C)** are shown for 20 minutes imaging period.

Supplemental Figure 4

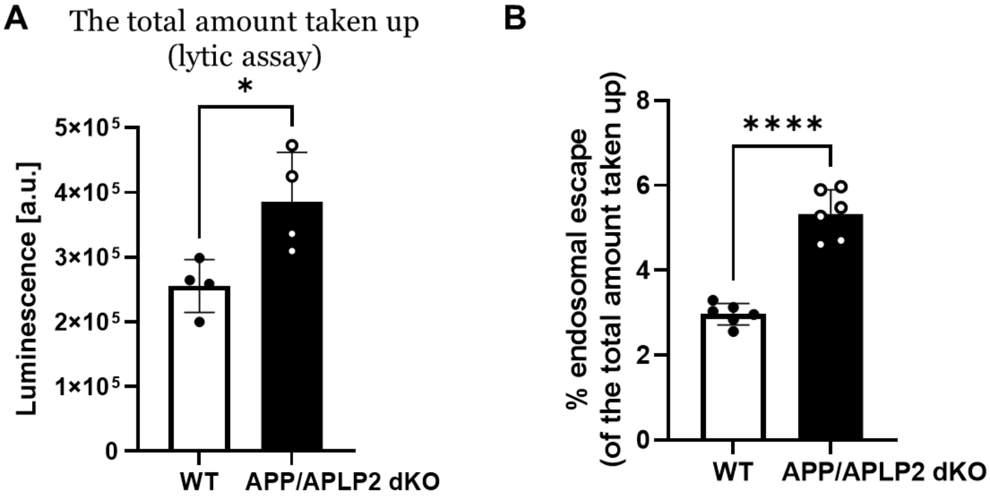

(A) APP/APLP2 dKO MEF cells or WT controls were incubated with 100 nM HiBiT-tau for 30 min, then the medium was washed out, and the cell lysates were subjected to the Nano-GloTM HiBiT Lytic Detection System, in which the amount of HiBiT-tau in cells can be determined by adding a lytic detection reagent containing the substrate and LgBiT. N = 4 independent biological samples. Unpaired t-test, *p<0.05 **(B)** The % endolysosomal escape was calculated by dividing the amount of escaped HiBiT-tau (Figure 2H) by the total intracellular HiBiT-tau levels (Supplemental Figure 4A)**,** suggesting that the tau entry process is significantly increased in APP/APLP2 dKO cells. Unpaired t-test, ****p<0.001

Supplemental Figure 5

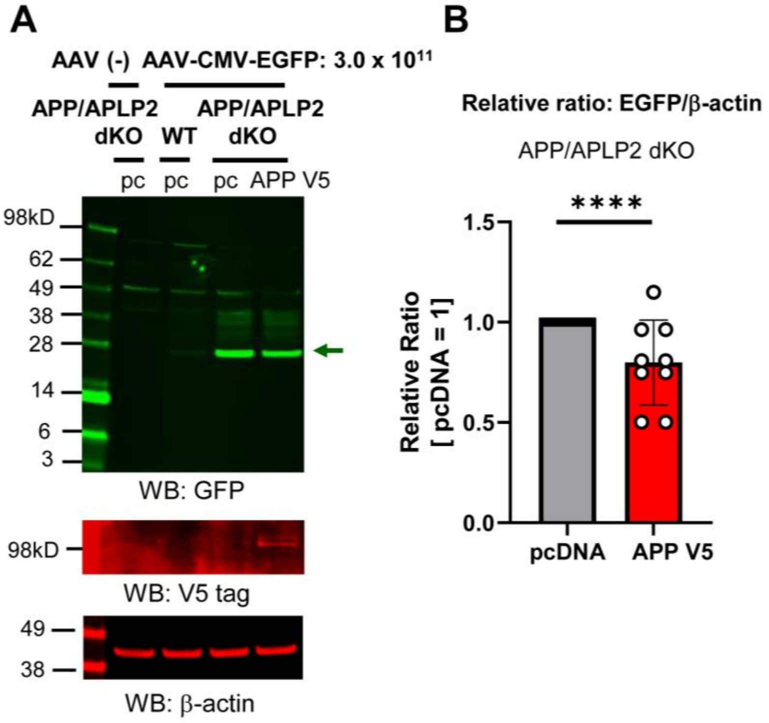

(A) APP/APLP2 dKO MEF cells were transfected either with empty vector (pcDNA3.1) or WT APP V5. 24 hours after transfection, the cells were incubated with AAV-CMV-EGFP (stereotype 8) (3.0 x 10^{11} GC) for 72 hours, followed by Western blotting using an anti-GFP antibody. β-Actin was used as a loading control. **(B)** The band quantification of EGFP over β-actin shows decreased EGFP expression in WT APP V5 transfected APP/APLP2 dKO cells compared to empty vector. N = 9 independent experiments. One-sample t-test. ****p<0.001

Supplemental Figure 6

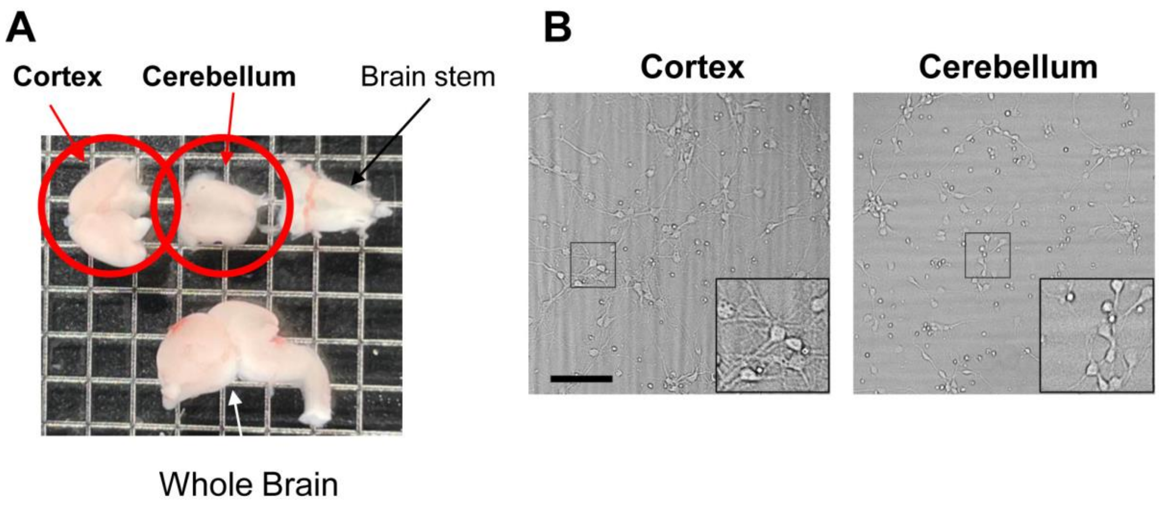

(A) Primary neurons were dissociated from the cortex or the cerebellum of mouse embryo and **(B)** cultured for 12-15 DIV. Scale bar 100 μm

Supplemental Figure 7

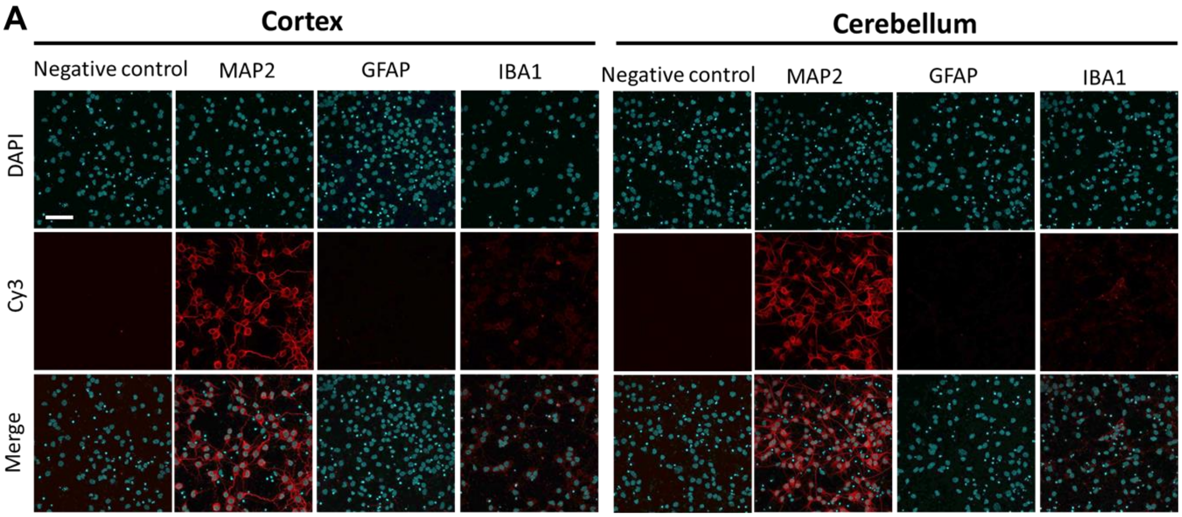

B

Cortex	DAPI (+)	MAP2 (+)
Image 1	85 neurons	78 (91.7%)
Image 2	60 neurons	59 (98.3%)
Image 3	77 neurons	71 (92.2%)

Cerebellum	DAPI (+)	MAP2 (+)
Image 1	139 neurons	109 (78.4%)
Image 2	140 neurons	132 (94.3%)
Image 3	151 neurons	134 (88.7%)

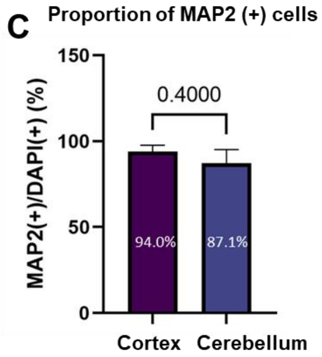

(A) Primary neurons from cortex and cerebellum were stained with MAP2 (neurons), GFAP (astrocytes), or IBA1 (microglia) and Cy3-conjugated secondary antibodies, followed by confocal microscopy. **(B)** The proportion of neurons in the culture was determined by calculating MAP2-positive over DAPI-positive cells in three independent images, **(C)** which was not different between the cerebellum and cortical neurons ($p = 0.40$), $N = 3$, unpaired t-test.

Supplemental Figure 8

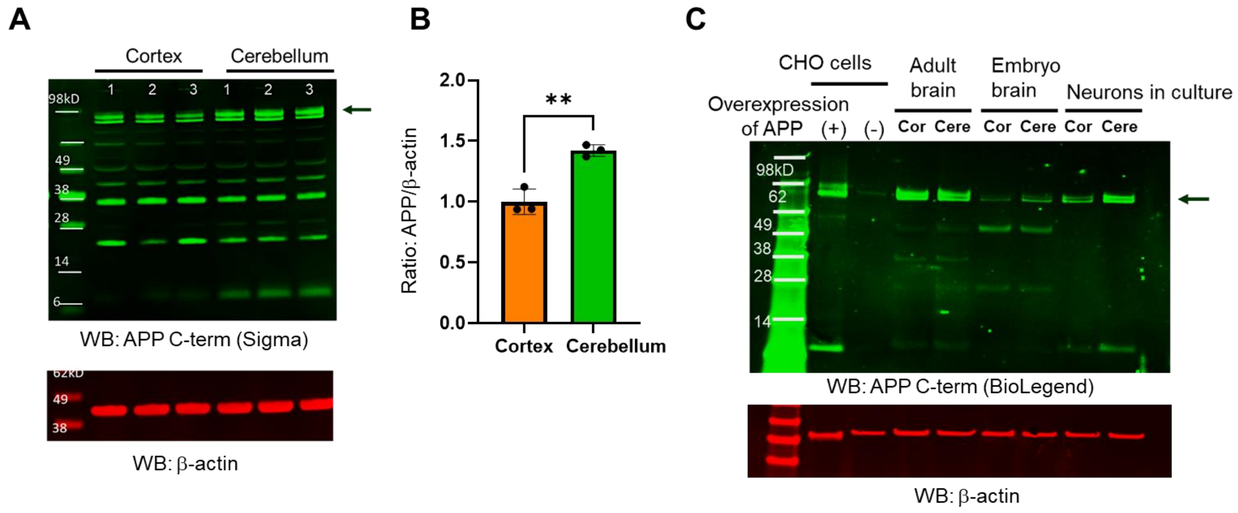

(A) Western blot analysis using an APP (rabbit polyclonal, Sigma) and β-actin (loading control) antibodies, **(B)** verifying significantly higher APP expression in the cerebellum than in the cortical primary neurons. N = 3, unpaired t-test, **p<0.01. **(C)** While APP expression is higher in cerebellum than cortical in primary neurons in culture and embryo brain lysates, it is comparable between the two brain regions of adult mouse brain lysates. CHO cells transiently transfected with APP was used as a positive control of APP detection.

Supplemental Figure 9

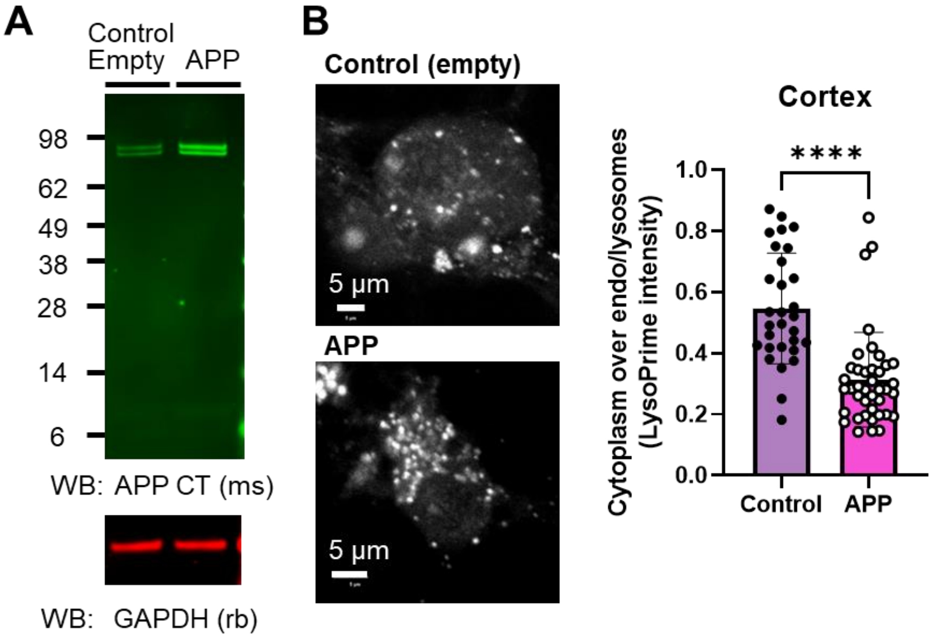

(A) Western blot analysis using an APP C-term (CT) and GAPDH antibodies, verifying significantly higher APP expression in the cortical primary neurons transduced with lentivirus packaging mouse APP (MOI 2.5, 5 days). **(B)** LysoPrime Green imaging showed that cytoplasmic over endo-lysosome intensity ratios in cortical neurons overexpressing APP are lower than those in neurons transduced with control virus post HNE treatment (300 µM, 30 min). N = 30-40 ROIs from 4-6 neurons, Mann-Whitney U tests, ****p<0.0001, scale bar 5 µm
